# Transferrin receptor levels and its rare variant are associated with human obesity

**DOI:** 10.1111/1753-0407.13467

**Published:** 2023-08-30

**Authors:** Jin Qiu, Zhiyin Zhang, Yepeng Hu, Yuhan Guo, Caizhi Liu, Yanru Chen, Dongmei Wang, Junlei Su, Sainan Wang, Mengshan Ni, Sainan Xu, Jian Yu, Tianhui Hu, Gaojie Song, Xinran Ma, Xuejiang Gu, Jiqiu Wang, Lingyan Xu

**Affiliations:** ^1^ Shanghai Key Laboratory of Regulatory Biology Institute of Biomedical Sciences and School of Life Sciences, East China Normal University Shanghai China; ^2^ Department of Endocrinology and Metabolism, China National Research Center for Metabolic Diseases Ruijin Hospital, Shanghai Jiao Tong University School of Medicine Shanghai China; ^3^ Department of Endocrine and Metabolic Diseases The First Affiliated Hospital of Wenzhou Medical University Wenzhou China

**Keywords:** brown gene program, genetic variants, obesity, *TFRC*, thermogenic adipocytes

## Abstract

**Aim:**

Iron homeostasis is critical for functional respiratory chain complex of mitochondrial, thus potentially contributing to fat biology and energy homeostasis. Transferrin receptor (*Tfrc*) binds to transferrin for extracellular iron uptake and is recently reported to be involved in brown fat development and functionality. However, whether *TFRC* levels and variants are associated with human obesity is unknown.

**Methods:**

To investigate the association of *TFRC* levels and variants with human obesity, fat biopsies were obtained from surgery. Exon‐sequencing and genetic assessments were conducted of a case–control study. For *TFRC* levels assessment in fat biopsy, 9 overweight and 12 lean subjects were involved. For genetic study, obese (*n* = 1271) and lean subjects (*n* = 1455) were involved. *TFRC* levels were compared in abdominal mesenteric fat of pheochromocytoma patients versus control subjects, and overweight versus lean subjects. For genetic study, whole‐exome sequencing of obese and matched control subjects were conducted and analyzed. In addition, the possible disruption in protein stability of *TFRC* variant was assessed by structural and molecular analysis.

**Results:**

*TFRC* levels are increased in human browning adipose tissue and decreased in fat of overweight patients. Besides, *TFRC* levels are negatively correlated with body mass index and positively correlated with uncoupling protein 1 levels. Furthermore, a rare heterozygous missense variant p.I337V in *TFRC* shows a tendency to enrich in obese subjects. Structural and functional study reveals impaired protein stability of the *TFRC* variant compared to wild‐type*.*

**Conclusions:**

Reduced *TFRC* levels and its rare variant p.I337V with protein instability are associated with human obesity.

## Graphical abstract

Highlights

- Evidence for contribution of transferrin receptor (*Tfrc*) and its variants for human thermogenic fat is limited. *TFRC* levels is elevated in activated human thermogenic fat from pheochromocytoma patients and downregulated in fat from overweight patients compared to their control subjects, respectively.
- Whole‐exome sequencing of obese and matched control subjects suggests that a rare heterozygous missense variant p.I337V in *TFRC* shows a tendency to enrich in young, severely obese subjects.
- Structural and functional studies reveal impaired protein stability of the *TFRC* variant p.I337V compared to wild‐type*.*

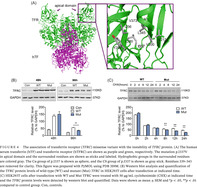

## 1 INTRODUCTION

Obesity is manifested as excess accumulation of fat in adipose tissues. Three classes of adipose tissues exist, including white adipose tissue as a major site for energy storage and brown adipose tissue with capability of thermogenesis and energy expenditure.^1^ Beige adipocytes, a newly discovered type of adipocytes, exist in clusters within certain white fat depots and morphologically resemble white adipocytes in rest state. Upon cold or β‐adrenergic activation, beige adipocytes are activated to possess a brown‐like appearance and high thermogenic capacity, a process referred to as “browning of white fat.”^2^, ^3^, ^4^, ^5^ Clinically relevant, functional brown and beige fat have recently been shown to exist in adults. Human deep cervical, supraclavicular, and paraspinal areas are demonstrated to be enriched with brown and beige adipocytes as a heterogenous mixture with varying constituents depending on their tissue depths.^6^ Besides, under physiological or pathological stimulation, white adipocytes in humans can also be induced to browning. For instance, the sustained activation of β3‐adrenergic signaling in pheochromocytoma patients causes strong white fat browning and enhanced thermogenesis, resulting a pathologically lean phenotype in these patients.^7^, ^8^ Critically, adipocytes undergo metabolic declines in the development of obesity or aging, leading to impaired beige/brown fat thermogenesis and reduced ability of white fat browning, indicating the potential of targeting adipose tissues to prevent and treat obesity or metabolic dysfunctions in humans.^4^, ^9^

Iron homeostasis has been shown to play vital roles in various physiological and pathological processes, including metabolic diseases, via molecular and clinical studies.^10^ For example, serum ferritin levels have been shown to associated with metabolic syndrome in multiple human cohorts.^11^ Recently, iron levels have been linked to fat biology.^12^ Genetic manipulation of genes related to iron homeostasis, such as transferrin receptor (*TFRC*), a membrane protein binding to transferrin for extracellular iron uptake, resulted in impaired adipogenesis and functionality of fat in mice and consequently affected their energy metabolism, suggesting the importance of iron metabolism in obesity and metabolic diseases.^13^, ^14^

In the present study, we sought to unveil the association of *TFRC* levels and *TFRC* variants with human obesity. Of note, we found that, in abdominal mesenteric regions of fat, *TFRC* levels were increased in pheochromocytoma patients and decreased in overweight patients, compared to their control counterparts. Besides, *TFRC* levels negatively correlated with body mass index (BMI) and positively correlated with uncoupling protein 1 (UCP1) levels in fat. In addition, via whole‐exome sequencing (WES), we discovered a rare heterozygous missense variant p.I337V in *TFRC*, which showed a tendency to enrich in obese subjects, indicating its potential contribution to obesity possibly due to its impaired protein stability. Overall, we revealed the association of *TFRC* with human obesity and proposed *TFRC* as a promising therapeutic target for obesity and metabolic diseases in human.

## 2 MATERIALS AND METHODS

### 2.1 Human adipose tissue biopsy samples

Human fat biopsies were obtained from the abdominal mesenteric region of fat from overweight (BMI ≥ 25 kg/m^2^) and lean subjects (18 kg/m^2^ < BMI < 25 kg/m^2^), as well as pheochromocytoma patients and control subjects who received surgery in the First Affiliated Hospital of Wenzhou Medical University or Ruijin Hospital, Shanghai Jiao Tong University School of Medicine (SJTUSM), with written informed consent, as described before.^15^

### 2.2 Immunostaining of adipose tissues

For immunohistochemistry staining, adipose tissues were placed in Bouin fixative, embedded in paraffin, and subsequently cut into 5 μm sections. Sections were deparaffinized hydrated in xylene and graded ethanol and rinsed in PBS for 5 min before being incubated with pepsin (Sigma, USA). The sections were incubated for 10 min in 0.3% H_2_O_2_ to quench endogenous peroxidase activity. Sections were blocked and incubated at 4°C overnight with diluted polyclonal antibodies against UCP1 (ab10983, Abcam, UK) and *TFRC* (ab84036, Abcam, UK) and with horseradish peroxidase conjected goat‐anti rabbit IgG for 1 h. DAB chromogen (DAB Substrate Kit, H‐2200, Vector Labs, USA) was used for peroxidase detection of immunoreactivity. Images were taken with a Zeiss 710 confocal microscope.

### 2.3 Human genetic study

For genetic analysis of low‐frequency or rare variants in the coding regions of *TFRC* gene with the threshold of minor allele frequency below 5% (MAF < 5%), we examined our in‐home database consisting of the WES data of patients with obesity (BMI ≥30 kg/m^2^) and matched control subjects.^16^ Basic information was obtained from the Genetics of Obesity in Chinese Youngs study, which was previously established by Ruijin Hospital and registered in ClinicalTrials.gov (ClinicalTrials reg. no. NCT01084967, http://www.clinicaltrials.gov/).^17^, ^18^ The clinical procedures for anthropometric assessment, biochemical measurement, blood glucose, and lipid ascertainment were performed according to previously described protocols.^17^ Functional prediction of *TFRC* variants is conducted by SIFT (https://sift.bii.a-star.edu.sg/), PolyPhen2 (http://genetics.bwh.harvard.edu/pph2/), and Mutation taster (http://mutationtaster.org).

### 2.4 Ethics statement

The human studies were approved by the Institutional Review Board of First Affiliated Hospital of Wenzhou Medical University and Ruijin Hospital, SJTUSM. A written informed consent was obtained from each subject.

### 2.5 Construction of *TFRC* wild‐type and variant plasmid and transfection in HEK293T cells with or without cycloheximide treatment

The *TFRC* coding sequences were obtained from cDNA and the polymerase chain reaction (PCR) primers or site mutation primers are listed in Supplementary Table S1. *TFRC* and mutant plasmids were transfected into HEK293T cells according to the manufacturer's protocol (C4058L1080, Shanghai Life iLab Biotech, China) and proteins were harvested at the indicated time. To further examine the protein stability of *TFRC* and its mutant, after transfection for 48 h, HEK293T cells were treated with cycloheximide (CHX; C7698, Sigma, USA) for the indicated time.

### 2.6 Mito‐tracker analysis

Mitochondria quality and membrane integrity were assessed by staining with MitoTracker® Red CMXRos (Thermo Fisher, United States). Immortalized beige adipocytes were differentiated to day 4 and overexpressed with wild‐type (WT) or mutant (Mut) *Tfrc*. After 48 h, mature adipocytes were incubated with 100 nM MitotTracker for 20 min and then washed three times with PBS. Cells were fixed with 4% PFA for 10 min and fluorescent images were obtained using the Nikon inverted microscope ECLIPSE Ts2.

### 2.7 Lentiviral constructions and infection

*Tfrc* and mutant *Tfrc* lentiviral particles were constructed and concentrated (GeneChem company, Shanghai). Immortalized beige preadipocytes were infected with lentiviral particles as follows: 2 × 10^9^ PFU lentivirus carrying WT and Mut *Tfrc* were added to cell culture medium with 4 mg/mL sequa‐brene (S2667, Sigma, USA) for 24 h and switched to fresh medium for another 3 days. Samples were collected for real‐time PCR and western blot analysis.

### 2.8 Real time PCR

RNAs from adipose tissues were extracted using Trizol (9109, Takara, Japan). Total RNA (1 μg) was reversely transcribed with PrimeScript RT reagent Kit with gDNA Eraser kit (PR047Q, Takara, Japan) and quantitative PCR was performed using SYBR green (11143ES50, Yeasen, China) on the LightCycler480 system (Roche, Switzerland). Messenger RNA (mRNA) levels were calculated by the ΔΔCT method with the level of 36B4 or Pcdh as the internal control. The primers used for quantitative PCR are in Supplementary Table S2.

### 2.9 Western blot

For western blot, the protein concentrations were quantified using a BCA Protein Assay kit (P0010, Beyotime Biotechnology, China) and equal amounts of protein were subjected to 10% sodium dodecyl sulfate polyacrylamide gel electrophoresis and then transferred to a nitrocellulose membrane (66 485, PALL, USA). After blocking with 5% skimmed milk, the membrane was incubated overnight at 4°C with indicated primary antibodies and subsequently incubated with secondary antibody at room temperature for 1 h. The images of the blots were detected using the Odyssey imaging system (LIed ovBiotechnology, USA).^19^ Antibodies used for western blot are as follows: anti‐TFRC (ab84036, Abcam, UK), anti‐GAPDH (sc‐32233, Santa Cruz, USA), IRDye 800CW Goat anti‐Rabbit IgG (P/N 926‐32211, LI‐COR Biotechnology, USA), and IRDye 800CW Goat anti‐Mouse IgG (P/N 926‐32210, LI‐COR Biotechnology, USA).

### 2.10 Statistical analysis

Data are presented as means ± SEM in molecular analysis, and means ± SD in human clinical parameters. All statistical analysis was performed with GraphPad Prism software. The rare missense frequency in *TFRC* was calculated using the chi‐square test, and two‐tailed Student's *t* test was used in other experiments to determine *p* values and *p* < .05 was considered as significant.

## 3 RESULTS

### 3.1 *TFRC* levels are increased in activated human thermogenic fat from pheochromocytoma patients

**FIGURE 1:**
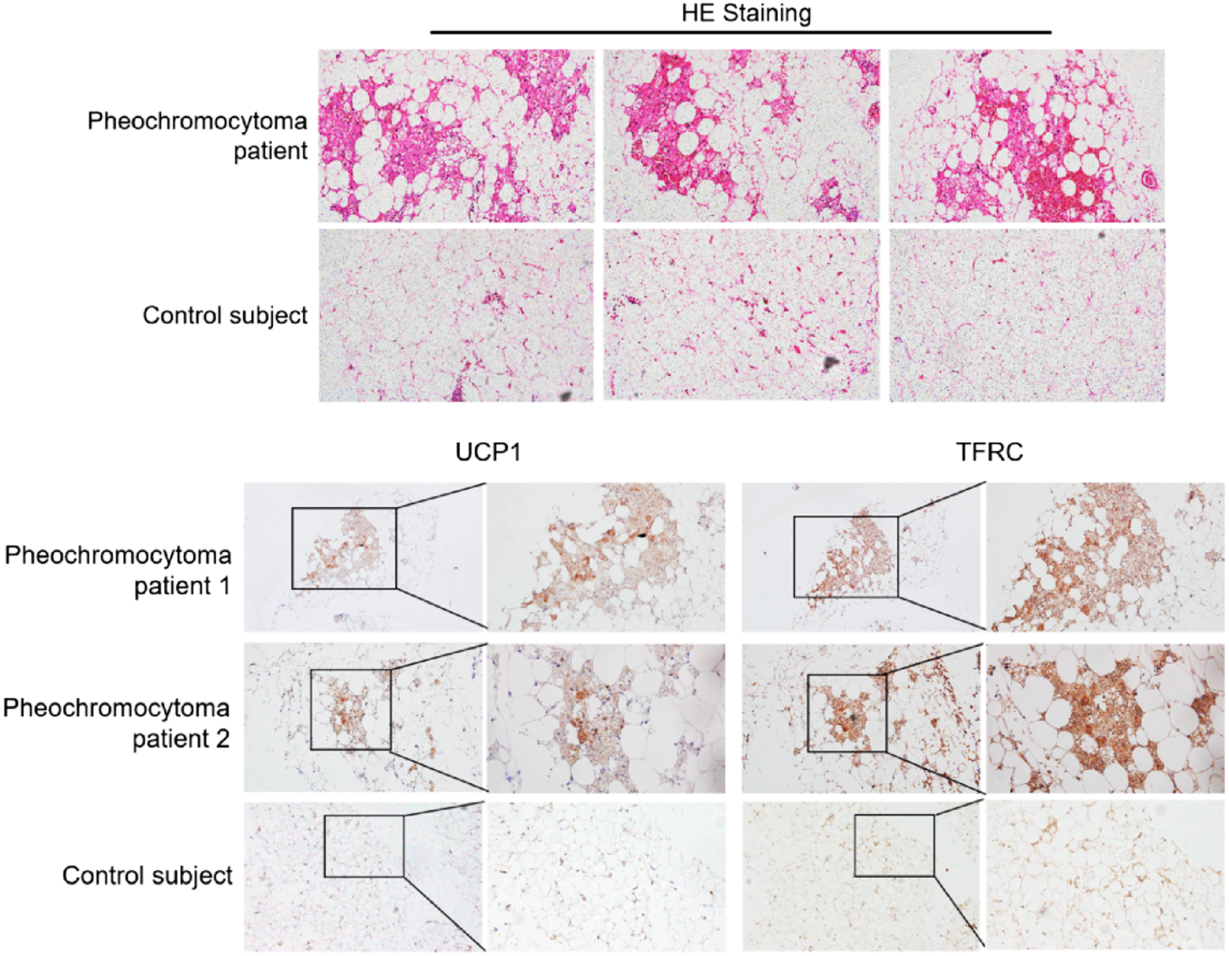
Transferrin receptor (*TFRC*) levels in activated human thermogenic fat from pheochromocytoma patients. (A) Representative HE staining of abdominal mesenteric fat from pheochromocytoma patients and control subjects. (B) Representative immunohistochemistry staining of UCP1 and *TFRC* in abdominal mesenteric fat from pheochromocytoma patients and control subjects. HE, hematoxylin and eosin; UPC1, uncoupling protein 1.

Pheochromocytoma patients are clinically characterized by abnormal high levels of catecholamine secretion. This results in an excess sympathetic tone and strongly induces white fat browning especially in patients with high catecholamines levels.^8^ We collected fat biopsies from pheochromocytoma patients and control subjects^15^ and validated enhanced white fat browning in the abdominal mesenteric regions of pheochromocytoma patients but not for controls (Figure 1A). Of clinical relevance, we found that *TFRC* levels were significantly increased in adipose tissues from pheochromocytoma patients compared to control subjects (Figure 1B). Notably, *TFRC* staining showed a similar expression pattern and intensity compared to UCP1, a major thermogenic marker (Figure 1B). Thus, these results suggested that *TFRC* levels in humans were correlated with thermogenic capacity in adipose tissues.

### 3.2 *TFRC* levels are decreased in adipose tissue of obese patients, as well as correlated with UCP1 levels and BMI

**FIGURE 2:**
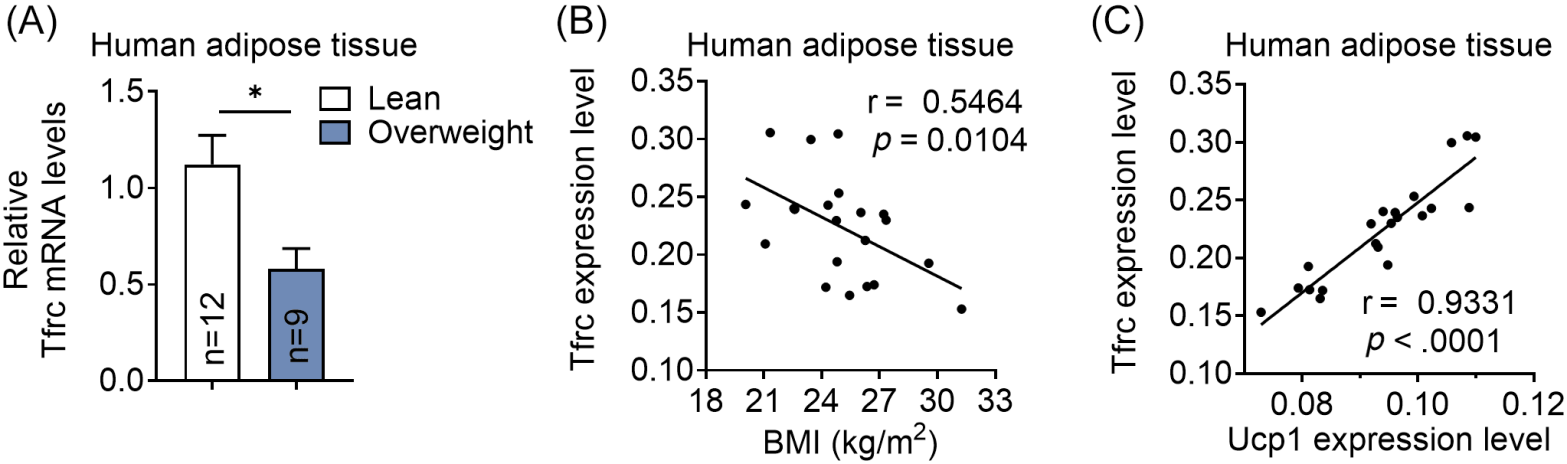
Transferrin receptor (*TFRC*) levels in human thermogenic fat. (A) *TFRC* levels in the omental fat of overweight subjects (*n* = 9), compared to lean control subjects (*n* = 12). (B) The correlation between *Tfrc* and BMI in human biopsies. (C) The correlation between *Tfrc* and Ucp1 mRNA expression levels in human biopsies. ΔCt values were used for analysis. BMI, body mass index; UPC1, uncoupling protein 1.

We next assess the association of *TFRC* levels with obesity in an overweight subjects (BMI ≥25 kg/m^2^, *n* = 9) and lean controls (18 kg/m^2^ < BMI < 24.9 kg/m^2^, *n* = 12) (Supplementary Table S3). Importantly, *TFRC* levels are decreased in abdominal mesenteric fat from overweight subjects compared to controls (Figure 2A). In addition, *TFRC* levels in fat are negatively correlated with BMI of human participates and positively correlated with thermogenic marker gene UCP1 levels in human biopsies (Figure 2B,C), suggesting the close correlation of *TFRC* levels with thermogenic adipocytes and obesity progression in human.

### 3.3 Rare nonsynonymous *TFRC* variants are enriched in young, severely obese subjects

**FIGURE 3:**
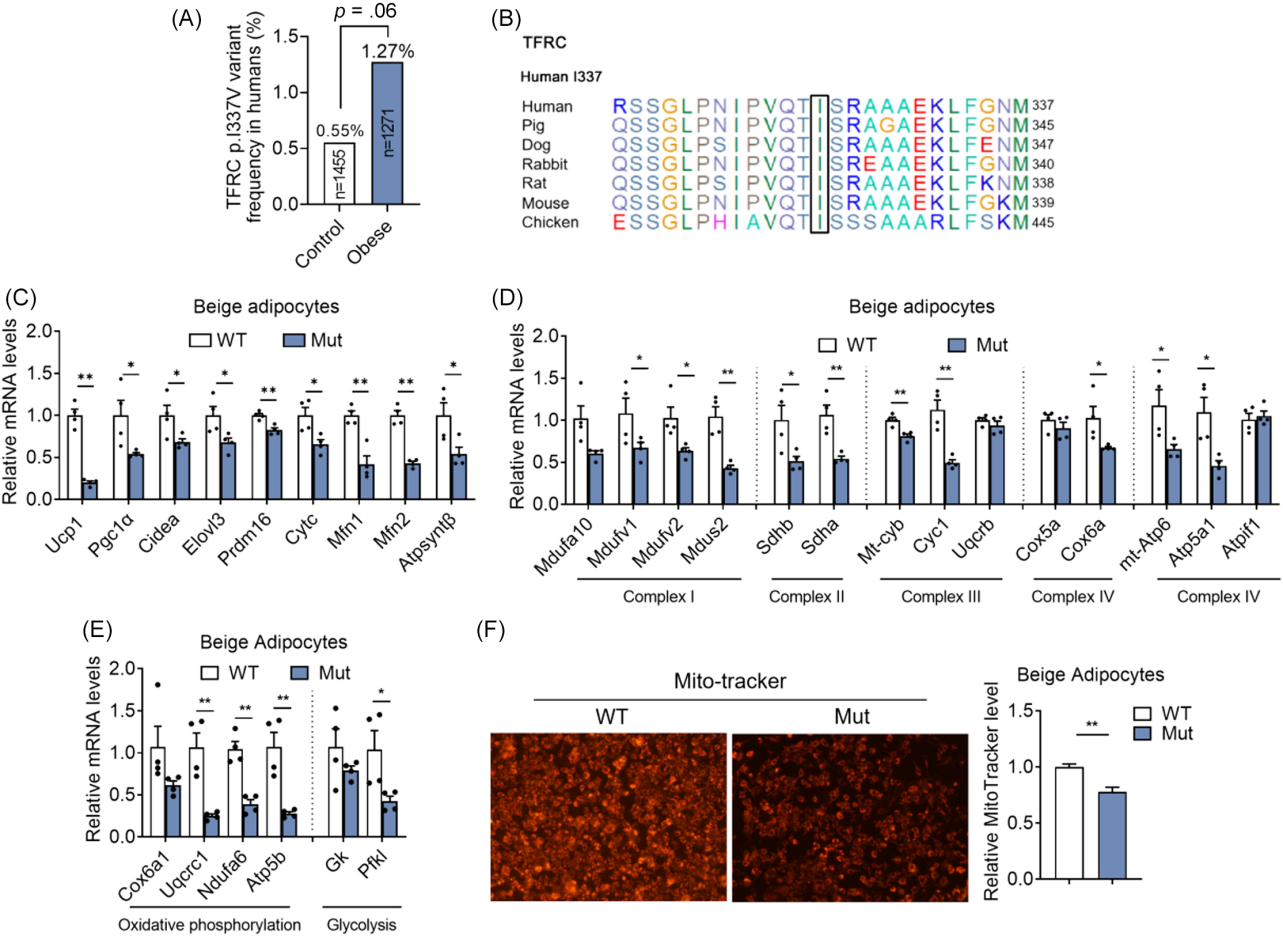
The correlation of transferrin receptor (*TFRC*) with human obesity. (A) Comparison of the frequency of the *TFRC* rare variants in control subjects and obese patients. (B) Sequence conservation analysis of the *TFRC* orthologs related to variants identified in obese subjects and controls. Mutant sites are shown with black box. (C–E) Gene expression analysis of thermogenic markers (C), mitochondrial respiratory chain complex markers (D), oxidative phosphorylation and glycolytic markers (E) from beige adipocytes overexpressed with wild‐type (WT) or mutant (Mut) *TFRC*. (F) Mito Tracker staining of mature beige adipocytes overexpressed with wild‐type (WT) or mutant (Mut) *TFRC*. Data were shown as mean ± SEM and *p < .05, **p < .01 compared to control group. mRNA, messenger RNA; UPC1, uncoupling protein 1.

We next examined whether *TFRC* variants/mutants exist in human and how they impact the development of human obesity. Previous genome‐wide association studies have identified the common variations nearby *TFRC* gene, with MAF >5%, are associated with low mean cell volume (MCV) of erythrocytes in Asian and European population.^20^, ^21^ A rare homozygous mutation of p.Y20H in *TFRC* was first reported in two pedigrees to cause low MCV and combined immunodeficiency by impaired *TFRC* internalization and consequent intracellular iron deficiency.^22^ To explore the potential nonsynonymous variants in *TFRC*, we screened the low‐frequency/rare variants (MAF <5%) in the gene in our in‐home database of WES data consisting of 1271 young, severely obese cases (age, 23.8 ± 7.4 years; BMI, 35.2 ± 4.7 kg/m^2^) and 1455 matched nonobese controls (Table 1).^16^ Of 23 coding mutations detected, 22 were rare missense mutations with MAF <1% except for p.L212V (MAF = 3%). Of note, a heterozygous p.I337V substitution in *TFRC* tended to be enriched in severely obesity group (odds ratio, 2.30; 95% confidence interval, 0.98–5.38; *p* = .06) (Figure 3A). Interestingly, the amino‐residue p.I337 is strictly conserved across different species with the exception of Pan troglodytes (Chimpanzee), which shows the same substitution with p.V337 as the rare mutation in carriers (Figure 3B). Of note, we overexpressed WT and Mut *Tfrc* in adipocytes and found decreased thermogenic and mitochondrial functions in mutant *Tfrc* overexpressed adipocytes, suggesting the impaired metabolic functionality of mutant *Tfrc* (Figure 3C,D). Indeed, Mut *Tfrc* overexpressed in mature beige adipocytes resulted in impaired mitochondrial quality and membrane integrity as revealed by MitoTracker staining when compared with WT *Tfrc* group (Figure 3E). We also compared the clinical features of obese carriers and noncarriers of the missense variant and found no substantial difference between the two groups in parameters related to metabolic disorders, including obesity traits, blood glucose, insulin sensitivity, islet β cell function, serum lipids, and liver enzymes (Supplementary Table S4).

**TABLE 1:** Rare and low‐frequency transferrin receptor (*TFRC*) nonsynonymous variants identified in young Chinese with obesity and lean controls.

Position^a^	rsID	Nucleotide change^b^	Amino acid change^b^	Cases	Controls	Case _freq	Control_freq	SIFT^§^	PolyPhen2^‡^_HDIV	Mutation taster^#^
chr3:195800925	rs201408488	T > C	T104A	1/1270	1/1454	0.0004	0.0003	T	B	T
chr3:195800829	‐	C > T	D136N	1/1270	0/1455	0.0004	‐	T	B	T
chr3:195798910	‐	C > A	R183L	1/1270	0/1454	0.0004	‐	T	B	D
chr3:195798320	rs41301381	G > C	L212V	1/77/1193	2/89/1364	0.0311	0.0320	T	B	T
chr3:195796384	‐	G > A	T248I	1/1270	4/1451	0.0004	0.0014	T	B	T
chr3:195794955	‐	A > G	M283T	0/1271	1/1454	‐	0.0003	T	P	D
chr3:195794438	‐	T > C	N331D	1/1270	0/1455	0.0004	‐	T	B	T
chr3:195794420	rs41295849	T > C	I337V	16/1255	8/1447	0.0063	0.0027	D	D	D
chr3:195794416	‐	G > C	S338C	1/1270	0/1455	0.0004	‐	D	D	D
chr3:195792457	‐	T > G	D352A	0/1271	2/1453	‐	0.0007	T	B	D
chr3:195792370	‐	A > C	L381R	1/1270	1/1454	0.0004	0.0003	T	P	D
chr3:195791240	rs41295879	C > T	G420S	1/1270	0/1455	0.0004	‐	T	B	D
chr3:195791192	‐	T > C	M436V	1/1270	0/1455	0.0004	‐	D	P	D
chr3:195787103	‐	T > C	K495R	1/1270	0/1455	0.0004	‐	T	B	D
chr3:195785491	‐	C > T	V517I	0/1271	1/1454	‐	0.0003	T	B	T
chr3:195785467	‐	C > T	D525N	1/1270	0/1455	0.0004	‐	T	P	D
chr3:195785461	‐	T > G	N527H	0/1271	1/1454	‐	0.0003	D	D	D
chr3:195782151	‐	C > T	G567S	0/1271	1/1454	‐	0.0003	T	P	D
chr3:195782058	rs144131234	C > T	V598M	1/1270	0/1455	0.0004	‐	T	B	D
chr3:195782043	‐	G > A	H603Y	1/1270	0/1455	0.0004	‐	T	B	D
chr3:195780362	‐	A > T	L656Q	1/1270	0/1455	0.0004	‐	D	D	D
chr3:195778910	rs202242239	G > A	T729M	3/1268	5/1450	0.0012	0.0017	D	D	D
chr3:195778820	‐	T > G	E759A	0/1271	1/1454	‐	0.0003	D	B	D

*Note*: Functional prediction is conducted by SIFT^§^ (https://sift.bii.a‐star.edu.sg/) (T, Tolerated; D, Deleterious), PolyPhen2^‡^ (http://genetics.bwh.harvard.edu/pph2/) (B, Benign; D, damaging; P, Probably), and Mutation taster^#^ (http://mutationtaster.org) (D, Damaging; T, Tolerated).

^a^ National Center for Biotechnology Information Build 37.

^b^ Variations are based on RefSeq records NM_001128148 and NP_001121620.

Abbreviation: Freq, allele frequency.

### 3.4 p.I337V substitution of *TFRC* shows impaired protein stability

**FIGURE 4:**
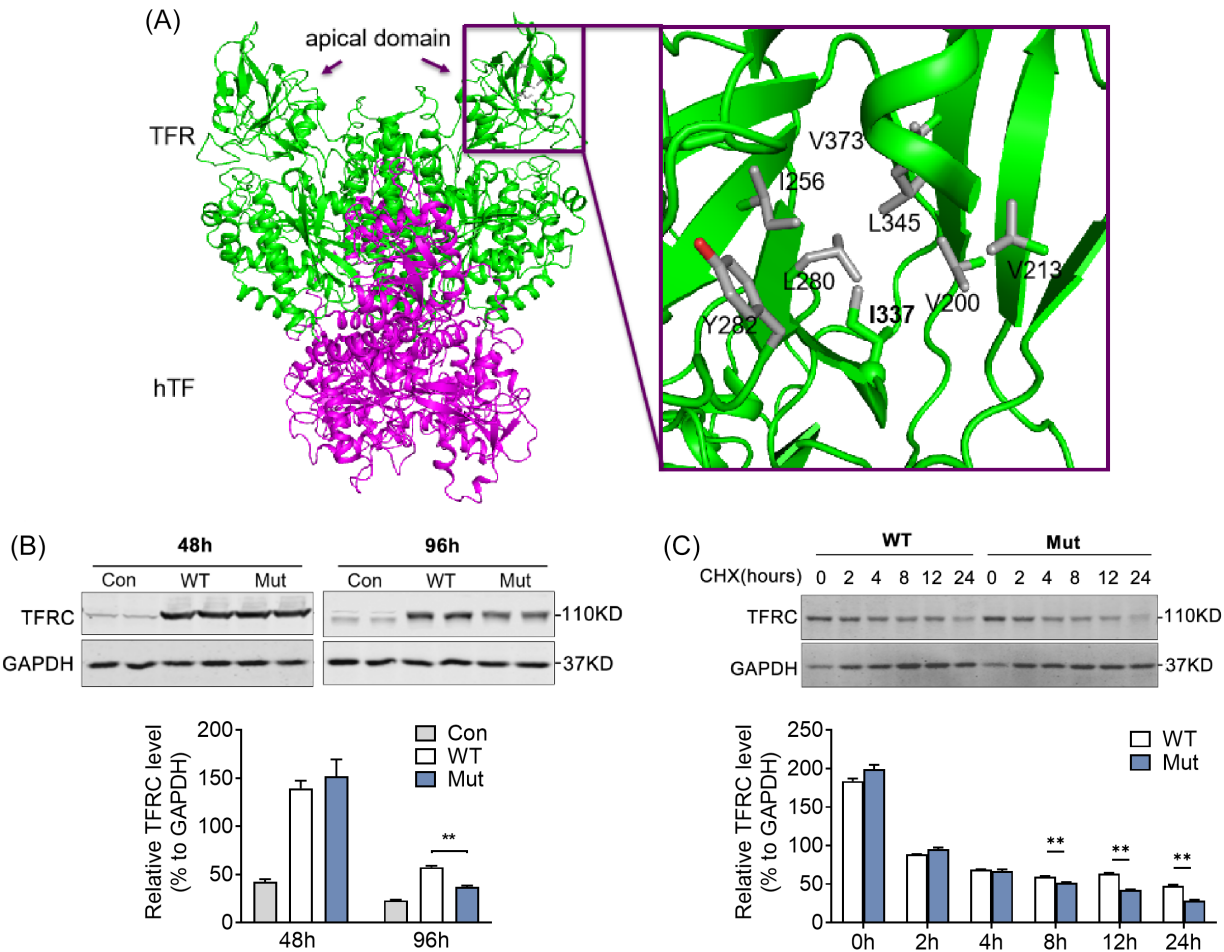
The association of transferrin receptor (*TFRC*) missense variant with the instability of *TFRC* protein. (A) The human serum transferrin (hTF) and transferrin receptor (hTFRC) are shown as purple and green, respectively. The mutation p.I337V in apical domain and the surrounded residues are shown as sticks and labeled. Hydrophobic groups in the surrounded residues are colored gray. The Cα group of p.I337 is shown as sphere, and the Cδ group of p.I337 is shown as gray stick. Residues 339–343 are removed for clarity. This figure was prepared with PyMOL using PDB 3S9M. (B) Western blot analysis and quantification of the *TFRC* protein levels of wild‐type (WT) and mutant (Mut) *TFRC* in HEK293T cells after transfection at indicated time. (C) HEK293T cells after transfection with WT and Mut TFRC were treated with 50 μg/mL cycloheximide (CHX) at indicated time and the *TFRC* protein levels were detected by western blot and quantified. Data were shown as mean ± SEM and *p < .05, **p < .01 compared to control group. Con, controls.

We further analyzed the potential structural change (PDB 3S9M)^23^ of the missense variant p.I337V and found that in WT p.I337 residue was surrounded by a group of hydrophobic residues and these residues form extensive van der Waals interactions in the core of the apical domain, whereas removal of a key alkyl group (CH2‐) as in the missense variant p.V337 may lead to instability of the hydrophobic core in the apical domain and compromise of *TFRC's* function (Figure 4A). Consistently, the missense variant was predicted to be functionally damaged by three types of predicting software (SIFT, PolyPhen2, and Mutation taster, Table 1). To verify this hypothesis, we constructed WT and Mut *TFRC* and transfected them in HEK293T cells to assess their protein stabilities. Indeed, we found that the *TFRC* Mut featured significantly reduced protein levels at 96 h, compared to WT *TFRC* (Figure 4B). The variants also showed decreased half‐time of *TFRC* when treated with protein synthesis inhibitor CHX (Figure 4C). These findings suggested a potential association of rare *TFRC* missense variant with the instability of *TFRC* protein and development of obesity in humans.

## 4 DISCUSSION

In the present study, we reported that *TFRC* was strongly expressed in abdominal fat tissue from pheochromocytoma patients and decreased in overweight patients. Besides, the rare variant p.I337V in *TFRC* was marginally associated with human obesity and the I337V amino acid substitution led to *TFRC* protein instability.

*Tfrc*, also known as Tfr1, plays an important role for cellular iron uptake and homeostasis as receptors for transferrin. Apotransferrin (iron‐free tranferrin) binds to iron atoms to form holotransferrin, which interacts with *Tfrc* and is internalized through receptor‐mediated endocytosis into acidified endosomes. Ferrous iron is then released into cytosol for subsequent iron (Fe) metabolism.^24^ Iron metabolism has attracted great attention recently because it is recently reported to be involved in several physiological and pathological processes, including hematopoiesis, immunity responses, cancers, and metabolic homeostasis.^25^, ^26^, ^27^ It has been reported that cellular iron deficiency caused by knocking out ferritin heavy/heart chain component of ferritin led to marked mitochondria dysfunction and reduction in energy expenditure and adaptive thermogenesis, suggesting the critical role of Fe metabolism in thermogenic adipocytes.^28^ Of note, *Tfrc* fat‐conditional knockout mice showed impaired thermogenesis, increased insulin resistance, and low‐grade inflammation accompanied by iron deficiency and mitochondrial dysfunction,^13^ which highly support our clinical findings that missense variant p.I337V of *TFRC* may affect *TFRC* protein stability, as well as decreased thermogenic and mitochondrial gene levels, thus increase obesity risk. Because *TFRC* is indispensable for cellular iron uptake, and considering the essential role of Fe‐S clusters for mitochondrial respiration, it is reasonable to postulate that an iron deficiency damaged mitochondrial thermogenesis function.^29^, ^30^, ^31^ Together, these studies and ours emphasized the critical role of intracellular iron balance in metabolic homeostasis in adipose tissues of rodents and humans.

It has been reported that browning of white adipose tissue occurs in pheochromocytoma patients due to the tumor‐mediated release of catecholamines.^7^, ^32^, ^33^ Besides, we and others have identified that *Tfrc* is expressed predominantly in thermogenic adipocytes versus white adipocytes, and its expression levels are tightly correlated with browning status.^13^, ^14^ Thus, the increased *TFRC* levels in adipose tissues from pheochromocytoma patients are likely due to the high browning status. It would be interesting to further investigate the possible mechanism that *TFRC* was regulated by β‐adrenergic signaling.

With genetic analysis of coding regions in *TFRC* gene, a rare missense variant p.I337V was identified to be enriched in obesity cases. The residue is evolutionally conserved across species from chicken to human only with the exception of chimpanzees, and the variant was predicted to be loss‐of‐function change by crystal structure analysis and predicting software. Whether the variant in those rare carriers was derived from chimpanzees or evolved separately remains to be explored in following studies.

In the present study, we have provided initial evidence supporting the association between *TFRC* levels and rare variants with human obesity. Future studies to validate the clinical relevance of missense variant p.I337V of *TFRC* with obesity need to be conducted in larger cohorts. Besides, it would be interesting to further explore the potential mechanisms underlying this relationship, as well as the detailed regulatory mechanism of *TFRC* protein instability. Further studies are warranted to potentially target *TFRC* signaling and iron metabolism in obesity treatment.

## 5 CONCLUSIONS

In summary, we identified that *TFRC* levels were increased in human browning adipose tissue and decreased in fat of obese patients. Besides, *TFRC* levels were positively correlated UCP1 levels in fat and negatively correlated with BMI in humans. Of note, *TFRC* rare missense variant p.I337V conservatively showed protein instability compared to WT *TFRC* in mice and humans. Overall, our study emphasized that *TFRC* rare variant may be associated with obesity development and *TFRC* gene may be a potential therapeutic target in treating human obesity.

## AUTHOR CONTRIBUTIONS

Lingyan Xu, Jiqiu Wang, and Xuejiang Gu conceived the project and designed the experiments. Jin Qiu, Zhiyin Zhang, and Yepeng Hu carried out most of the experiments and collected human biopsies. Yuhan Guo, Yanru Chen, Junlei Su, and Caizhi Liu assisted in data analysis. Dongmei Wang, Sainan Wang, Sainan Xu, and Tianhui Hu provided rodent biological samples for association analysis. Gaojie Song assisted with *TFRC* structure analysis. Xinran Ma, Lingyan Xu, Jiqiu Wang, and Xuejiang Gu wrote and edited the paper.

## CONFLICT OF INTEREST STATEMENT

No potential conflicts of interest relevant to this article were reported.

## Supporting information


**Supplementary Table S1.** Polymerase chain reaction (PCR) primers for constructions of transferrin receptor (*TFRC*) plasmid.
**Supplementary Table S2.** Primer sequences for quantitative real‐time polymerase chain reaction (qRT‐PCR).
**Supplementary Table S3.** Baseline characteristics of lean and overweight subjects.
**Supplementary Table S4.** The clinical parameters related to obesity in transferrin receptor (*TFRC*) p.I337V variant obese carriers and noncarriers.

## Data Availability

The data sets used during the current study are available from the corresponding authors upon reasonable request.
