# Supplementary material for: Transferrin receptor levels and its rare variant are associated with human obesity
Source: J Diabetes. 2023 Aug 30;16(1):e13467. doi: 10.1111/1753-0407.13467 (PMC10809288; doi:10.1111/1753-0407.13467)
Supplement: Supplementary file 1 — Supplementary Table S1. Polymerase chain reaction (PCR) primers for constructions of transferrin receptor (TFRC) plasmid. Supplementary Table S2. Primer sequences for quantitative real‐time polymerase chain reaction (qRT‐PCR). Supplementary Table S3. Baseline characteristics of lean and overweight subjects. Supplementary Table S4. The clinical parameters related to obesity in transferrin receptor (TFRC) p.I337V variant obese carriers and noncarriers. [file JDB-16-e13467-s001.docx]

**Supplementary Table 1** PCR primers for constructions of TFRC plasmid.

| **Primer Name** | **Sequences 5'-3'** |
| --- | --- |
| TFRC-WT-F | CCGGAATTCATGATGGATCAAGCCAGATCAGCA |
| TFRC-WT-R | CGCGGATCCTTAAAACTCATTGTCAATATTCCAAATGTC |
| TFRC-Mut-F | CCTAATATACCTGTGCAAACAGTGTCAAGAGCTGCTGCAGAAAAG |
| TFRC-Mut-R | CTTTTCTGCAGCAGCTCTTGACACTGTTTGCACAGGTATATTAGG |

**Supplementary Table** **2** Primer sequences for quantitative real-time PCR (qRT-PCR).

| **Gene name** | **Forward primer sequence 5'-3'** | **Reverse primer sequence** **5'-3'** |
| --- | --- | --- |
| hGapdh | GAAGGTGAAGGTCGGAGT | GAAGATGGTGATGGGATTTC |
| hTfrc | GGCTACTTGGGCTATTGTAAAGG | CAGTTTCTCCGACAACTTTCTCT |
| hUcp1 | GGCTACTTGGGCTATTGTAAAGG | CAGTTTCTCCGACAACTTTCTCT |
| Pcdh | CTGCAATGATACCGCGAGAC | AATAGACTGGATGGAGGCGG |
| m36b4 | GCTTCATTGTGGGAGCAGAC | ATGGTGTTCTTGCCCATCAG |
| mTfrc | GGCTACTTGGGCTATTGTAAAGG | CAGTTTCTCCGACAACTTTCTCT |
| mUcp1 | GGCCCTTGTAAACAACAAAATAC | GGCAACAAGAGCTGACAGTAAAT |
| mPgc1α | ACCATGACTACTGTCAGTCACTC | GTCACAGGAGGCATCTTTGAAG |
| mCidea | TGACATTCATGGGATTGCAGAC | CGAGCTGGATGTATGAGGGG |
| mElovl3 | TTCTCACGCGGGTTAAAAATGG | TCTCGAAGTCATAGGGTTGCAT |
| mPrdm16 | CCACCAGCGAGGACTTCAC | GGAGGACTCTCGTAGCTCGAA |
| mCpt1α | TTGCCCTACAGCTCTGGCATTTCC | GCACCCAGATGATTGGGATACTGT |
| mCox4β | CTGCCCGGAGTCTGGTAATG | CAGTCAACGTAGGGGGTCATC |
| mCytc | AAATCTCCACGGTCTGTTCGG | GGGTATCCTCTCCCCAGGTG |
| mMfn1 | AACCGAGAAGCTGCAGATGA | AGTTGGGCCACATCACACTC |
| mMfn2 | AGGCCTTCCTCCTCACAGAG | GCAGGGTCAGTCAGGTCATCA |
| mATPsyntβ | GACATGGGCACAATGCAGG | GCAGGGTCAGTCAGGTCATCA |

**Supplementary Table 3** Baseline characteristics of Lean and Overweight subjects.

| **Characteristics** | **Lean** | **Overweight** | ***P* value** |
| --- | --- | --- | --- |
|  | (n=12) | (n=9) |  |
| Age (years) | 48.67 ± 14.29 | 58.67 ± 13.79 | 0.124 |
| Sex |  |  | 0.367 |
| Male, *n* (%) | 6 (50.0%) | 2 (22.2%) |  |
| Fmale, *n* (%) | 6 (50.0%) | 7 (77.8%) |  |
| Height (cm) | 165.75 ± 7.28 | 160.11 ± 6.45 | 0.081 |
| Weight (Kg) | 63.92 ± 6.81 | 70.22 ± 7.08 | 0.053 |
| Body temperature (℃) | 36.74 ± 0.26 | 36.87 ± 0.26 | 0.290 |
| Heart rate (beats/min) | 86.17 ± 30.14 | 81.78 ± 17.48 | 0.702 |
| SBP (mmHg) | 125.58 ± 9.75 | 133.56 ± 8.78 | 0.068 |
| DBP (mmHg) | 81.00 ± 5.26 | 82.22 ± 8.18 | 0.681 |
| Blood glucose (mmol/L) | 6.24 ± 1.20 | 7.26 ± 1.97 | 0.197 |
| Chronic disease |  |  |  |
| T2DM, *n* (%) | 0 (0.0%) | 2 (22.2%) | 0.171 |
| Hypertension, *n* (%) | 5 (41.7%) | 5 (55.6%) | 0.670 |

Abbreviation: SBP: systolic blood pressure; DBP: diastolic blood pressure; T2DM: type 2 diabetes mellitus. Data are means ± SD, and *n* (%).

**Supplementary Table 4** The clinical parameters related to obesity in TFRC p.I337V variant obese carriers and non-carriers.

| **Clinical parameters** | **p.I337V carriers** | | **p.I337V non-carriers** | | **P value** |
| --- | --- | --- | --- | --- | --- |
|  | N | Mean ± SD | N | Mean ± SD |  |
| Age (years) | 15 | 24.33 ± 6.08 | 315 | 24.27 ± 5.75 | 0.965 |
| Birthweight (g) | 9 | 3183.33 ± 555.09 | 264 | 3389.91 ± 581.45 | 0.295 |
| Height (m) | 13 | 1.68 ± 0.11 | 305 | 1.71 ± 0.09 | 0.180 |
| Weight (kg) | 12 | 95.28 ± 13.89 | 307 | 103.48 ± 17.85 | 0.117 |
| BMI (kg/m^2^) | 14 | 34.13 ± 3.03 | 305 | 35.22 ± 4.36 | 0.353 |
| WC (cm) | 13 | 107.81 ± 14.00 | 300 | 111.19 ± 12.15 | 0.330 |
| HC (cm) | 12 | 113.75 ± 9.01 | 299 | 115.39 ± 9.00 | 0.535 |
| WHR | 12 | 0.96 ± 0.20 | 297 | 0.96 ± 0.08 | 0.756 |
| HbA1c (%) | 6 | 7.07 ± 3.36 | 200 | 5.89 ± 1.23 | 0.432 |
| FPG (mmol/l) | 13 | 5.24 ± 0.58 | 295 | 5.48 ± 1.41 | 0.539 |
| 2hPG (mmol/l) | 13 | 8.53 ± 2.27 | 294 | 8.09 ± 3.17 | 0.620 |
| FPI (μIU/ml) | 12 | 18.02 ± 8.41 | 288 | 23.18 ± 13.68 | 0.197 |
| 2hPI (μIU/ml) | 12 | 149.06 ± 105.92 | 288 | 145.61 ± 118.41 | 0.921 |
| HOMA-IR | 12 | 4.18 ± 2.10 | 288 | 5.68 ± 3.76 | 0.171 |
| HOMA-B | 12 | 234.71 ± 141.81 | 288 | 290.83 ± 195.72 | 0.327 |
| TG (mmol/l) | 9 | 4.08 ± 5.22 | 257 | 2.09 ± 2.35 | 0.285 |
| TC (mmol/l) | 9 | 5.40 ± 1.82 | 256 | 4.74 ± 1.01 | 0.308 |
| HDL-c (mmol/l) | 9 | 1.08 ± 0.26 | 256 | 1.06 ± 0.24 | 0.853 |
| LDL-c (mmol/l) | 9 | 3.03 ± 1.22 | 257 | 2.93 ± 0.83 | 0.821 |
| ALT (IU/L) | 11 | 59.36 ± 56.90 | 267 | 59.03 ± 48.52 | 0.982 |
| AST (IU/L) | 11 | 35.45 ± 27.70 | 268 | 33.63 ± 23.77 | 0.804 |
| GGT (IU/L) | 11 | 25.91 ± 17.84 | 267 | 37.25 ± 31.74 | 0.241 |
